# Short and Long Non-Coding RNAs in Renal Cell Carcinoma

**DOI:** 10.3390/ncrna12020008

**Published:** 2026-02-27

**Authors:** Monia Cecati, Valentina Pozzi, Valentina Schiavoni, Giuseppina Barrasso, Veronica Pompei, Daniela Marzioni, Nicoletta Bonci, Stefania Fumarola, Andrea Ballini, Davide Sartini, Roberto Campagna

**Affiliations:** 1 Department of Human Sciences and Promotion of the Quality of Life, San Raffaele Roma University, 00166 Rome, Italy; monia.cecati@uniroma5.it; 2 Department of Clinical Sciences, Polytechnic University of Marche, 60126 Ancona, Italy; v.pozzi@univpm.it (V.P.); s1104554@studenti.univpm.it (G.B.); v.pompei@univpm.it (V.P.); d.sartini@univpm.it (D.S.); 3 Department of Experimental and Clinical Medicine, Polytechnic University of Marche, 60126 Ancona, Italy; d.marzioni@univpm.it (D.M.); s1119807@studenti.univpm.it (N.B.); 4 Advanced Technology Center for Aging Research, Istituto di Ricovero e Cura a Carattere Scientifico (IRCCS) INRCA, 60121 Ancona, Italy; s.fumarola@inrca.it; 5 Department of Life Science, Health and Health Professions, Link Campus University, 00165 Rome, Italy; a.ballini@unilink.it

**Keywords:** renal cell carcinoma, miRNAs, lncRNAs, circRNAs

## Abstract

Renal cell carcinoma (RCC) represents the most frequent kidney malignancy and remains a major clinical challenge due to its often silent onset, high metastatic potential, and limited responsiveness to conventional chemotherapy. Increasing evidence indicates that non-coding RNAs (ncRNAs), including microRNAs (miRNAs), long non-coding RNAs (lncRNAs), and circular RNAs (circRNAs), are key regulators of RCC tumorigenesis, progression, and therapy resistance. Rather than providing a purely descriptive overview, this review focuses on emerging mechanistic paradigms through which ncRNAs actively shape tumor behavior and therapeutic response in RCC. This review summarizes current knowledge on the biological and clinical relevance of ncRNAs in RCC, highlighting their dual roles as oncogenic drivers or tumor suppressors through the modulation of pathways involved in proliferation, apoptosis, angiogenesis, invasion, immune evasion, metabolic reprogramming, and ferroptosis. Particular emphasis is placed on mechanistically defined ncRNA regulatory axes controlling ferroptosis, autophagy, metabolic reprogramming, and immune escape, as well as on ncRNA-mediated intercellular communication via extracellular vesicles, which promotes the dissemination of resistance to targeted therapies. The review also addresses ncRNA-based diagnostic and prognostic applications, including miRNA signatures capable of discriminating RCC subtypes and circulating ncRNAs as minimally invasive biomarkers. Moreover, the manuscript discusses ncRNA-mediated mechanisms of resistance to targeted therapies such as sunitinib, sorafenib, and axitinib, emphasizing regulatory networks involving miRNA targets, lncRNA–miRNA sponging, RNA-binding proteins, extracellular vesicle transfer, and epigenetic modulation. Emerging therapeutic opportunities are also addressed, including strategies aimed at inhibiting oncogenic ncRNAs or restoring tumor-suppressive ncRNAs to enhance drug sensitivity and improve patient stratification.

## 1. Introduction

Renal cell carcinoma (RCC) is the most common malignancy arising in the kidney and accounts for about 90% of newly diagnosed kidney cancers [1]. Its incidence and mortality have been increasing globally, and the disease is reported more frequently in men than in women [2]. In particular, men between 60 and 70 years of age often develop more aggressive tumors, which are typically associated with higher stage and grade [3]. RCC develops from the proximal renal tubular epithelium and includes three main histological subtypes: clear cell (ccRCC), papillary (pRCC), and chromophobe (chRCC). Among these, ccRCC is the predominant subtype, representing roughly 80% of cases, and it accounts for most RCC-related deaths [4]. This variant shows a marked tendency to metastasize, with common sites of spread including the lungs [5], liver [6], bones [7], and lymph nodes [8]. At the time of first diagnosis, distant metastases are already present in approximately 30% of ccRCC patients, likely because the early phases of the disease are often clinically silent [9]. RCC occurrence increases with advancing age, but several lifestyle-related factors also contribute to its development; obesity, alcohol consumption, hypertension, and cigarette smoking are all associated with higher risk.

Surgical resection remains the gold standard for patients with early-stage RCC [10]. However, because a considerable proportion of individuals present with metastatic disease at diagnosis or develop metastases after treatment, there is a strong need to expand research efforts toward additional therapeutic strategies. In patients with inoperable RCC, targeted therapies represent a key option and can improve overall survival as well as delaying disease progression [11]. In this context, treatments aimed at blocking regulatory pathways that support metastatic dissemination may represent particularly effective approaches for advanced, non-resectable disease. Immunotherapy, especially immune checkpoint blockade, together with targeted therapy, most notably using antiangiogenic agents such as monoclonal antibodies, kinase inhibitors, and inhibitors of the mechanistic target of rapamycin (mTOR) and vascular endothelial growth factor (VEGF) pathways, has emerged as a promising therapeutic avenue [12]. RCC tumorigenesis is a multifactorial and dynamic process driven by genetic and epigenetic alterations that disrupt cellular homeostasis and promote uncontrolled growth [13]. Among the molecular mechanisms involved, DNA methylation changes, histone modifications, genomic instability, and driver gene mutations have all been implicated in RCC progression [14].

As with several malignant and non-malignant disorders [15,16,17,18,19,20], RCC can also arise from inherited conditions; loss of chromosome 3p, a region containing tumor suppressor genes such as Von Hippel–Lindau (VHL), SET domain-containing 2, histone lysine methyltransferase (SETD2), Polybromo 1 (PBRM1), and BRCA1-associated protein 1 (BAP1), has been linked to RCC pathogenesis [21]. Alterations affecting VHL, including its inactivation, represent one of the most frequent genetic events observed in RCC [22]. The VHL gene encodes a protein involved in controlling the hypoxia-inducible factor (HIF) pathway; therefore, loss of VHL leads to increased expression of HIF-regulated genes that support glucose metabolism, cell proliferation, and angiogenesis [23].

Given that RCC is often detected at advanced stages, displays a strong propensity for metastasis, and shows limited sensitivity to conventional chemotherapy, the identification of new biomarkers is a major priority. Such biomarkers could support earlier diagnosis, refine prognostic assessment, and contribute to the development of novel targeted treatments for more effective clinical management [24,25,26].

While dysregulation of protein-coding genes is clearly relevant, increasing evidence indicates that non-coding RNAs (ncRNAs) also contribute substantially to key oncogenic processes in RCC [27]. ncRNAs are transcribed RNA molecules that do not encode proteins and include several subclasses, such as long non-coding RNAs (lncRNAs), microRNAs (miRNAs), circular RNAs (circRNAs), and small nuclear RNAs [28]. Multiple studies have suggested that altered ncRNA expression is not only linked to RCC initiation and progression but also to therapy resistance, influencing fundamental cellular functions including proliferation, migration, apoptosis, and metabolic reprogramming [29].

The purpose of this review is to examine the contribution of ncRNAs to RCC development and to discuss their involvement in mechanisms of drug resistance. Unlike previous reviews that have primarily focused on individual classes of non-coding RNAs, this review provides a comprehensive and integrated overview of miRNAs, lncRNAs, and circRNAs in renal cell carcinoma. By jointly examining these ncRNA classes, we highlight their interconnected regulatory networks and emerging mechanistic roles in tumor progression, immune modulation, and resistance to targeted therapies. This integrated perspective aims to frame ncRNAs as active drivers of disease biology rather than isolated molecular markers.

## 2. MicroRNAs, Long Non-Coding RNAs and Circular RNAs

**Figure 1:**
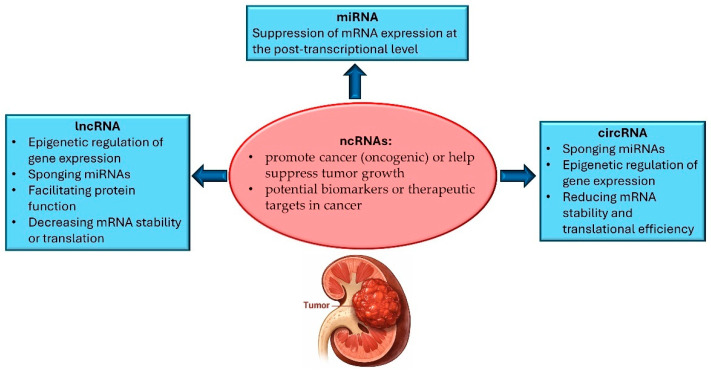
Roles of non-coding RNAs in renal cell carcinoma. Schematic overview showing how major classes of non-coding RNAs (ncRNAs)—long non-coding RNAs (lncRNAs), microRNAs (miRNAs), and circular RNAs (circRNAs)—regulate gene expression and influence tumor biology. lncRNAs and circRNAs modulate oncogenic pathways through epigenetic regulation, miRNA sponging, effects on protein function, and alterations in mRNA stability/translation, while miRNAs primarily suppress mRNA expression at the post-transcriptional level.

RCC is defined by deregulated cell proliferation, the capacity to invade and disseminate to distant sites, and the disruption of physiological cell death programs. In recent years, ncRNAs have been identified as important regulators of multiple cellular processes [30,31,32,33]. This discovery has driven strong research interest, as ncRNAs represent a highly diverse group of transcripts, including molecules with oncogenic activity as well as others with tumor-suppressive functions [34]. Consequently, several clinical trials are currently investigating ncRNAs as cancer biomarkers and as potential therapeutic targets. The recognition of ncRNAs as key components of gene regulation has provided additional layers of understanding of cancer biology, clarifying how these molecules contribute to tumor initiation and progression and how they may be exploited therapeutically. In particular, dysregulated ncRNA expression and the downstream signaling networks they affect have been strongly associated with malignant transformation and disease progression [35]. Moreover, genetic alterations affecting loci that encode ncRNAs have been linked to several tumor types [27]. ncRNAs include miRNAs, lncRNAs, and circRNAs (Figure 1).

miRNAs are small RNAs, approximately 22 nucleotides in length, that regulate gene expression by binding to complementary sequences, most commonly within the 3′ untranslated region (3′UTR) of target mRNAs. This interaction typically results in mRNA degradation and/or inhibition of translation. Altered miRNA expression has been reported across multiple cancer types and has been shown to influence key cellular and molecular programs involved in malignant transformation. Although the full spectrum of miRNA functions in cancer is still being defined, miRNAs are known to act either as tumor suppressors or as oncogenic regulators, thereby contributing to tumor growth, progression, and metastatic dissemination. Individual miRNAs are able to regulate multiple targets simultaneously and can modulate the expression of both protein-coding and non-coding transcripts. Their targets include oncogenes such as rat sarcoma proto-oncogene (RAS), MYC proto-oncogene (MYC), and epidermal growth factor receptor (EGFR), as well as tumor suppressors including tumor protein p53 (TP53), phosphatase and tensin homolog (PTEN), and BRCA1. A well-known example of an oncogenic miRNA is miR-155, which has been implicated in aberrant B-cell proliferation and the development of leukemia and lymphoma. Similarly, miR-21 is frequently upregulated in pre-B cell lymphoma and has also been associated with several other malignancies. Conversely, several miRNAs exert tumor-suppressive functions. For instance, the let-7 family can downregulate RAS expression, thereby limiting oncogenic signaling. miR-15a and miR-16-1 also act as tumor suppressors; however, their mutation or deletion has been linked to chronic lymphocytic leukemia. Finally, miR-34a regulates multiple oncogenic pathways and has been reported to be inactivated in certain tumors, including prostate cancer and multiple myeloma [27].

lncRNAs are RNA transcripts longer than 200 nucleotides that regulate gene expression through multiple mechanisms, including epigenetic modulation, chromatin remodeling, transcriptional control, post-transcriptional regulation, and signal transduction. lncRNAs have been widely implicated in cancer initiation, progression, and metastatic dissemination [36]. lncRNAs can exert their functions in cis, influencing nearby genes close to their transcriptional locus, or in trans, acting on distant genomic regions. In addition, several lncRNAs facilitate molecular interactions by bringing together regulatory components such as mRNAs, miRNAs, and proteins, including transcription factors, RNA-binding proteins (RBPs), and chromatin-modifying complexes, thereby serving as scaffolds that support the formation of functional regulatory assemblies [37]. For instance, metastasis-associated lung adenocarcinoma transcript 1 (MALAT1) has been reported to increase the expression of enhancer of zeste homolog 2 (EZH2) by interacting with miR-205 and to promote apoptosis in acute lymphoblastic leukemia cells. Overall, multiple studies indicate that lncRNAs can contribute to tumor development by behaving as oncogenic regulators [38]. HOX transcript antisense RNA (HOTAIR) is a well-characterized oncogenic lncRNA that is frequently upregulated in breast cancer and has been linked to unfavorable clinical outcomes [39]. However, not all lncRNAs promote tumorigenesis; several exert protective roles by limiting proliferation, triggering apoptosis, preserving genomic integrity, or sustaining tumor suppressor pathways. Maternally expressed gene 3 (MEG3) is among the best-studied lncRNAs with tumor-suppressive properties. It has been reported to enhance p53 protein levels and to modulate the transforming growth factor beta (TGFβ) signaling pathway, ultimately reducing cell proliferation [40,41]. Another established tumor-suppressive lncRNA is GAS5, which is often downregulated across different cancer types and has been validated in vivo in glioblastoma and breast cancer models. In addition, GAS5 can influence glucocorticoid receptor signaling and interact with miRNAs involved in the control of cell growth, promoting apoptosis and reducing migratory potential [42].

circRNAs are produced through back-splicing of precursor mRNA transcripts and are characterized by a covalently closed loop structure. This configuration makes circRNAs highly stable in vivo, supporting their potential utility as biomarkers and, in some cases, as therapeutic targets or tools [43].

Beyond their oncogenic or tumor-suppressive functions, ncRNAs can also contribute to the emergence of drug resistance in cancer cells. Several preclinical investigations have explored ncRNA-based strategies to overcome therapy resistance. For example, the lncRNA plasmacytoma variant translocation 1 (PVT1) has been reported to promote gemcitabine resistance in pancreatic cancer by activating the Wingless-related integration site (Wnt)/β-catenin axis and autophagy-related pathways. Accordingly, PVT1 has been proposed as a potential therapeutic target, as its downregulation may restore gemcitabine sensitivity. In breast cancer, MALAT1 has been shown to be upregulated in drug-resistant cells, and its inhibition can increase responsiveness to chemotherapy. Conversely, certain lncRNAs appear to counteract chemoresistance: long intergenic non-protein coding rna (LINC)00968 has been reported to reduce drug resistance in breast cancer by repressing Wnt2 and inhibiting Wnt2/β-catenin signaling, through a mechanism involving the recruitment of Hes-related family bHLH transcription factor with YRPW motif 1 (HEY1) [44].

Luo et al. reported that the lncRNA EIF3J divergent transcript (EIF3J-DT) is upregulated in gastric cancer cells with acquired resistance to chemotherapy. Their findings, supported by both in vitro and in vivo experiments, indicated that EIF3J-DT promotes autophagy and increases the expression of the autophagy-related 14 (ATG14) gene in resistant gastric cancer cells [45].

Among ncRNAs involved in chemosensitivity and chemoresistance, miRNAs are some of the most extensively investigated. A representative example is miR-125, which has been associated with therapy resistance across multiple tumor types. In breast cancer, miR-125 contributes to paclitaxel resistance by suppressing the pro-apoptotic factor Bcl-2 antagonist killer 1 (Bak1). It has also been shown to reduce the expression of dihydrofolate reductase (DHFR) and thymidylate synthase (TS), thereby promoting resistance to methotrexate or Tomudex in colon cancer and osteosarcoma. In contrast, miR-125b levels have been reported to inversely correlate with 5-fluorouracil resistance in hepatocellular carcinoma. Given these context-dependent effects, further preclinical investigation of anti-miRNA approaches is required before they can be advanced into clinical trials [46].

More recently, circRNAs have also been implicated in the regulation of chemoresistance. In osteosarcoma, hsa_circ_0001258 has been shown to increase glutathione s-transferase mu 2 (GSTM2) expression by sequestering miR-744-3p, thereby contributing to drug resistance. In addition, hsa_circ_0004015 has been reported to influence resistance to tyrosine kinase inhibitors (TKIs) in non-small cell lung cancer by modulating the miR-1183/3-phosphoinositide dependent protein kinase 1 (PDPK1) signaling axis. Overall, similarly to lncRNAs and miRNAs, circRNAs represent potential therapeutic targets for strategies aimed at limiting chemoresistance [46].

## 3. miRNAs in RCC

### 3.1. miRNAs Used for Diagnosis and Prognosis in RCC

Differential diagnosis in RCC remains a common challenge in routine pathology practice, and the identification of reliable biomarkers is therefore essential to improve diagnostic accuracy and patient management. miRNAs have potential clinical value in RCC diagnosis and may help differentiate among histological subtypes. Huang et al. compared miRNA expression profiles in 11 paired RCC specimens, consisting of ccRCC samples and their matched non-tumor renal tissues. Using microarray analysis, they identified a panel of 81 miRNAs, including 48 that were associated with ccRCC. Within this group, 17 miRNAs were downregulated in ccRCC compared with non-tumor tissue, 2 were upregulated, and 14 showed no significant differences between tumor and normal samples [47].

Fridman et al. reported overlapping miRNA expression patterns between oncocytoma and chromophobe RCC, as well as between ccRCC and pRCC. By analyzing miRNAs extracted from 71 formalin-fixed, paraffin-embedded renal tumor specimens, they developed a two-step decision-tree classifier based on the expression of six miRNAs. In the first step, miR-210 and miR-221 were used to separate the two main subtype pairs (clear cell vs. papillary and oncocytoma vs. chromophobe). In the second step, miR-200c and miR-139-5p enabled discrimination between oncocytoma and chromophobe tumors, whereas miR-31 and miR-126 were applied to distinguish clear-cell from papillary RCC [48].

In a separate study, Petillo et al. reported subtype-specific miRNA signatures that could serve as distinctive markers across RCC variants. They evaluated miRNA expression in 20 tumor samples, with four specimens per group: pRCC, chRCC, oncocytoma, ccRCC with favorable prognosis (overall survival > 5 years), and ccRCC with poor prognosis (overall survival < 5 years). Notably, increased expression of miR-424 and miR-203 in ccRCC allowed discrimination from the papillary subtype. In addition, miR-203 showed reduced expression in oncocytoma but was upregulated in chromophobe RCC, further supporting its potential value in differential diagnosis [49].

Overall, these studies indicate that miRNA expression profiling can discriminate not only between non-tumor and tumor renal tissue but also among distinct histological subtypes of renal cancer by capturing subtype-specific differences in the abundance of these short non-coding RNAs. As shown in the studies by Fridman [48] and Petillo [49], different miRNAs can have similar roles, suggesting that more studies are needed to identify all miRNAs involved in the different histological subtypes of RCC. Comparing miRNA expression patterns between normal and tumor renal tissues can support diagnostic improvement and provide insight into the biological functions of miRNAs in RCC. In this context, Juan et al. identified 35 miRNAs capable of distinguishing ccRCC from patient-matched normal kidney tissue with high accuracy. Among these, 26 miRNAs were consistently downregulated, whereas 9 were upregulated in ccRCC relative to normal samples. Notably, miR-155 and miR-21, both frequently overexpressed in several cancer types, as well as miR-210, a hypoxia-inducible miRNA, were also found to be increased in ccRCC. In addition, several of the downregulated miRNAs were associated with recurrent chromosomal deletions that are characteristic of ccRCC [50].

To support RCC diagnosis, miRNA profiling can also be applied to liquid biopsy samples, offering the advantage of a minimally invasive approach. Dias et al. investigated plasma-derived miRNA profiles in 32 patients with ccRCC sampled before and after surgery, as well as in 37 patients with metastatic disease. They reported that miR-25-3p, miR-126-5p, miR-200c-3p, and miR-301a-3p decreased after tumor resection, whereas miR-1293 levels increased. In addition, miR-301a-3p was elevated in patients with metastatic disease, while miR-1293 showed reduced expression in this group [51].

Multiple studies have shown that specific miRNA signatures may provide prognostic information and help predict survival outcomes in RCC. Yu-Zheng et al. identified 147 miRNAs capable of distinguishing tumor tissue from normal renal tissue. Among these, 22 miRNAs were significantly associated with overall survival: 13 were negatively correlated with survival (miR-223, miR-365-2, miR-21, miR-18a, miR-183, miR-335, miR-149, miR-9-2, miR-365-1, miR-130b, miR-9-1, miR-625, and miR-146b), whereas 9 miRNAs (miR-584, miR-10b, miR-27b, miR-769, miR-181a-2, miR-23b, miR-204, miR-24-1, and miR-139) were associated with more favorable survival outcomes [52]. In addition, Zhao et al. and Huang et al. reported further prognostic miRNAs. Zhao et al. found that reduced miR-497 expression correlated with advanced disease stage, higher histological grade, and metastatic spread, and was therefore linked to shorter overall survival. Using the Cancer Genome Atlas (TCGA) database, Huang et al. showed that increased expression of miR-21-5p, miR-223-3p, and miR-365a-3p was associated with worse survival in patients with ccRCC [53,54].

### 3.2. miRNAs and Drug Resistance in RCC

Drug resistance represents a major limitation in the treatment of many cancers and can markedly affect patient outcomes [55,56,57,58,59]. A substantial body of evidence supports the involvement of miRNAs in the resistance of RCC to targeted therapies (Table 1). For instance, miR-96-5p has been reported to be upregulated in sunitinib-resistant ccRCC, where it contributes to resistance by targeting PTEN and suppressing its expression [60].

miR-99a-3p has also been implicated in sunitinib resistance, as it targets the oncogene ribonucleotide reductase regulatory subunit M2 (RRM2) and promotes apoptosis in resistant cells. Consistently, reduced miR-99a-3p expression in RCC has been associated with poorer patient survival [54,61].

Thrombospondin 1 (THBS1), a gene linked to ccRCC response to sunitinib and patient prognosis, has been identified as a target of both miR-96-5p and miR-29b-3p. Quantitative reverse transcription polymerase chain reaction (qRT-PCR) analyses showed that miR-96-5p and miR-29b-3p were increased in ccRCC tumor tissues compared with matched adjacent non-tumor kidney samples. In parallel, decreased THBS1 expression in tumor tissue correlated with advanced stage, higher Fuhrman grade, and unfavorable prognosis. These results suggest that upregulation of miR-96-5p and miR-29b-3p may drive THBS1 suppression, thereby contributing to sunitinib resistance in ccRCC. Overall, these findings provide mechanistic insight into acquired drug resistance and may support the development of improved therapeutic strategies [62].

Sekino et al. reported, based on qRT-PCR analysis, that miR-130b expression was increased in RCC tissues compared with matched normal renal samples. They also showed that miR-130b influences cell growth and identified an inverse relationship between miR-130b levels and expression of the tumor suppressor PTEN in RCC. In particular, in vitro experiments supported a role for miR-130b in sunitinib resistance through PTEN regulation: miR-130b was significantly upregulated in sunitinib-resistant Caki-1 cells compared with the corresponding parental Caki-1 line [63].

miR-885-5p levels were reported to decrease significantly following sunitinib exposure, and this reduction was associated with unfavorable disease progression in ccRCC. Both in vitro and in vivo data supported a functional role for miR-885-5p suppression in the acquisition of sunitinib resistance. Mechanistically, sunitinib treatment was shown to reduce GATA binding protein 1 (GATA1) expression, leading to diminished GATA1 binding to the miR-885 promoter and consequent transcriptional repression of miR-885-5p. In addition, perilipin 3 (PLIN3) was validated as a direct target of miR-885-5p. PLIN3 participates in lipid droplet formation, a process that can limit oxidative stress by sequestering toxic lipids and thereby protecting solid tumor cells from lipotoxic damage. The authors found that PLIN3 upregulation occurred as a consequence of miR-885-5p downregulation induced by sunitinib treatment. Collectively, these findings suggest that the GATA1/miR-885-5p/PLIN3 regulatory axis may represent both a potential therapeutic target and a biomarker to predict and improve response to sunitinib in ccRCC [64].

miR-31-5p was identified as a key factor in promoting resistance to sorafenib in RCC cell lines. miR-31-5p has been identified as an important mediator of sorafenib resistance in RCC cell models. Although sorafenib can improve survival in patients with advanced RCC, its clinical benefit is often limited by the emergence of acquired resistance. Extracellular vesicles (EVs) are known to participate in intercellular communication by transporting bioactive molecules between cells. In this study, EVs released from sorafenib-resistant RCC cell lines (786-O and ACHN) were shown to be internalized by sorafenib-sensitive parental cells, inducing a resistant phenotype.

Among the EV cargo, miR-31-5p was enriched and was found to suppress MutL homolog 1 (MLH1), a gene implicated in drug responsiveness. Both in vitro and in vivo experiments confirmed that miR-31-5p promotes sorafenib resistance through MLH1 downregulation. Consistently, MLH1 expression was reduced in resistant cells, and re-expression of MLH1 restored sorafenib sensitivity. From a clinical perspective, patients who experienced disease progression during sorafenib therapy showed increased levels of miR-31-5p in circulating EVs compared with pre-treatment samples. Overall, these results indicate that EV-mediated transfer of miR-31-5p contributes to the spread of resistance within the tumor by targeting MLH1, highlighting miR-31-5p and MLH1 as potential biomarkers and therapeutic targets to counteract sorafenib resistance in RCC [65].

A recent study investigated VHL-dependent miRNAs in ccRCC. In particular, loss of function of the VHL tumor suppressor gene is recognized as one of the earliest and foundational events in the development of ccRCC. The resulting accumulation of HIFα due to VHL inactivation contributes to tumor progression, in part by modulating miRNA expression. miRNA expression profiling and high-throughput analyses were performed to identify a subset of miRNAs regulated by VHL in ccRCC. Among them, miR-2355-5p was found to be significantly upregulated in both ccRCC cell lines and patient tumor samples. Additionally, circulating levels of miR-2355-5p were markedly elevated in plasma from ccRCC patients. Further investigation revealed that the overexpression of miR-2355-5p is regulated by Hypoxia-inducible factor 2 alpha (HIF-2α). Functional studies using CRISPR/Cas9-mediated knockout of miR-2355-5p demonstrated impaired angiogenic potential, reduced cell proliferation, and significant suppression of tumor growth in mouse xenograft models. Through a miR-2355-5p pulldown assay, five tumor suppressor genes—Aconitase 1 (ACO1), BTG anti-proliferation factor 2 (BTG2), CKLF-like MARVEL transmembrane domain-containing 4 (CMTM4), slit guidance ligand 2 (SLIT2), and WD repeat and FYVE domain-containing 2 (WDFY2)—were identified as direct targets. These genes were consistently downregulated in both human ccRCC tumors and xenograft tissues. Overall, this study highlights the oncogenic role of miR-2355-5p and provides insight into its contribution to tumor angiogenesis and growth in VHL-deficient ccRCC [66].

miR-210-3p has been found to be downregulated in doxorubicin-resistant RCC cells (Caki-2/DOX). It enhances drug sensitivity by targeting the 3′UTR of ATP-binding cassette subfamily C member 1 (ABCC1), leading to reduced expression of multidrug resistance-associated protein 1 (MRP1), an efflux transporter associated with chemoresistance [67].

Another study reported that miR-124 is upregulated in renal cancer cells and contributes to cisplatin resistance. Specifically, miR-124 was shown to reduce cisplatin sensitivity in RCC mainly by inhibiting Calpain 4 (CAPN4). CAPN4 can promote the degradation of CCR4-NOT transcription complex subunit 3 (CNOT3), a key component of the CCR4–NOT transcriptional complex. CNOT3 has been implicated in the regulation of necroptosis by controlling the stability and translation of specific mRNAs.

Experimental evidence indicated that the increase in miR-124 observed after cisplatin exposure limits CAPN4 activity, thereby preventing CAPN4-dependent CNOT3 degradation and ultimately suppressing necroptosis in RCC cells. These results suggest that interfering with this regulatory axis could represent a therapeutic strategy to overcome cisplatin resistance in RCC by restoring alternative, non-apoptotic cell death programs [68].

## 4. Circular Non-Coding RNAs in Renal Cell Carcinoma

circRNAs are small closed-loop RNAs without 5′ caps or 3′ polyadenylate tails [69]. Originally, circRNAs were considered mere byproducts of transcription. Then, it was discovered that the majority of these were involved in both physiological and pathological processes, among which were cancer development and progression. circRNAs have been shown to serve multiple roles, including acting as sponges for microRNAs, binding molecules for RNA binding proteins (RBPs), regulators of transcription, or templates for protein synthesis. Their involvement in various types of tumors has been extensively studied. The functions of circRNAs are closely linked to their location within the cell: those found in the nucleus may play a role in controlling gene transcription and alternative RNA splicing [70], while cytoplasmic circRNAs commonly function as microRNA sponges or interact with RBPs to influence post-transcriptional regulation of genes associated with tumors [71]. Although circRNAs are often introduced as miRNA sponges, studies in RCC indicate that circRNA–miRNA interactions operate through defined and mechanistically relevant regulatory axes [72]. In RCC, circRNAs such as circASAP1, circGRAMD4, circ_0003520, circPDHK1, and circAGAP1 have been shown to modulate tumor growth, immune evasion, ferroptosis, and drug response by selectively binding specific miRNAs and thereby regulating downstream target genes. For example, circ_0003520 regulates RCC progression through the circ_0003520/miR-205-5p/Cullin 4B (CUL4B) axis, while circGRAMD4 modulates immune-related pathways by sponging miRNAs involved in antigen presentation [73,74]. Similarly, circAGAP1 influences therapeutic sensitivity through interaction with miRNAs targeting platelet-derived growth factor receptor (PDGFR) signaling [75]. These RCC-specific examples demonstrate that circRNA–miRNA interplay is not a generic sponge effect but a structured layer of post-transcriptional regulation that shapes oncogenic and tumor-suppressive pathways in a context-dependent manner.

Despite these insights, the specific functions of circRNAs in RCC are still not well understood (Table 2). Wang et al. revealed that circASAP1 was upregulated in ccRCC with a direct correlation with poor prognosis and metastasis. Moreover, the high expression of circASAP1 correlated with ccRCC cell viability, invasion and metastasis. Finally, they demonstrated the relationship between circRNA and the ArfGAP with SH3 domain, ankyrin repeat and PH domain 1 (ASAP1)/heterogeneous nuclear ribonucleoprotein C (HNRNPC)/glutathione peroxidase 4 (GPX4) axis. HNRNPC bound circASAP1 through amino acid residues 16–87. Silencing circASAP1 did not affect HNRNPC mRNA levels but led to a reduction in its protein levels via the ubiquitin–proteasome degradation pathway. Transcriptomic analysis revealed that protein-coding genes responsive to circASAP1 knockdown, including GPX4, were enriched in the ferroptosis pathway. Mechanistically, HNRNPC was found to regulate GPX4 expression by stabilizing its mRNA, thereby influencing its protein levels. Loss of circASAP1 expression induced a ferroptosis phenotype in RCC cells and significantly reduced both subcutaneous tumor growth and lung metastasis in nude mice. The decrease in circASAP1 expression suppresses RCC progression and metastasis by promoting ferroptosis through enhanced ubiquitination of HNRNPC. This highlights a novel regulatory axis and presents a potential therapeutic avenue for renal cancer treatment [76].

Zhou et al. revealed that high levels of circGRAMD4 in RCC correlated with a poor patient prognosis. Loss of circGRAMD4, due to the activation of CD8+ T cell-mediated anti-tumor immunity, led to a marked suppression of RCC proliferation. In particular, it was demonstrated that circGRAMD4 bound to RNA-binding motif protein 4 (RBM4) protein, enhancing the stability of NBR1 mRNA, an autophagic cargo receptor. This stabilization increased NBR1 expression, which promoted the degradation of major histocompatibility complex class I (MHC-1) molecules via macroautophagy/autophagy pathways. Consequently, antigen presentation in RCC cells was impaired, causing dysfunction of cluster of differentiation 8 positive (T lymphocyte) (CD8+) T cells and enabling tumor immune evasion. Combining circGRAMD4 inhibition with immune checkpoint blockade (ICB) reshaped the immunosuppressive tumor microenvironment, improving ICB therapeutic efficacy. Targeting circGRAMD4 could be a promising strategy to boost immunotherapy outcomes in combination with ICB [74].

circ_0003520 is another circRNA that is increased in ccRCC. It was demonstrated that circ_0003520 acts as an oncogene that drives ccRCC progression by modulating the miR-205-5p/CUL4B axis, highlighting a potential therapeutic target for inhibiting ccRCC growth. In vitro studies showed that knockdown of circ_0003520 inhibited cell proliferation, migration, invasion, and angiogenesis, and promoted apoptosis. Mechanism analysis revealed that circ_0003520 upregulated CUL4B through sequestering miR-205-5p. Inhibiting miR-205-5p or overexpressing CUL4B could reverse the suppressive effects of circ_0003520 knockdown on ccRCC. Additionally, circ_0003520 was shown to facilitate tumor growth in vivo through the miR-205-5p/CUL4B pathway. Overall, circ_0003520 acts as an oncogene that drives ccRCC progression by modulating the miR-205-5p/CUL4B axis, highlighting a potential therapeutic target for inhibiting ccRCC growth [73].

Ning et al. studied a possible delivery strategy to target circPDHK1, which is overexpressed in ccRCC and promotes cancer cell proliferation and migration. They developed a new nucleic acid drug delivery system, aptamer-guided lipid nanoparticle carrying siRNA against circPDHK1 (AS1411/LNP-si circPDHK1). Both in vitro and in vivo experiments showed that treatment with AS1411/LNP-si circPDHK1 led to a reduced expression of circPDHK1 in ccRCC cells. This delivery system demonstrated enhanced tumor-targeting efficiency and extended circulation time compared to non-targeted formulations. Importantly, AS1411/LNP-si circPDHK1 effectively blocked phosphorylation within the mTOR–protein kinase B (AKT) signaling pathway, inhibited proliferation and migration of ccRCC cells, and showed minimal toxicity in vital organs [77].

Lv et al. identified a marked upregulation of circAGAP1 in ccRCC, correlating with poor prognosis. In this study, they conducted a comprehensive investigation combining bioinformatics analyses and experimental validation to elucidate the function of circAGAP1 in sunitinib response. It was revealed that circAGAP1 is upregulated in sunitinib-sensitive ccRCC cells and enhances sensitivity to sunitinib by inhibiting cell proliferation, clonogenicity, and migration. Mechanistically, circAGAP1 acts as a competing endogenous RNA, sponging miR-149-5p, miR-455-5p, and miR-15a-5p, thereby modulating the expression of PDGFR, a known target of sunitinib. Overexpression of these miRNAs counteracted the sensitizing effect of circAGAP1, further validating this regulatory axis. circAGAP1 plays a critical role in modulating sunitinib sensitivity in ccRCC and may serve as a novel biomarker and potential therapeutic target to overcome drug resistance [75].

## 5. Long Non-Coding RNA in Renal Cell Carcinoma

lncRNAs can regulate different biological process, including cell proliferation, cell death, cell differentiation and cell cycle regulation, and they play pivotal roles in oncogenesis. A large number of lncRNAs have been related to RCC development and progression or participate in the resistance of RCC to targeted therapy (Table 3). Indeed, different studies have demonstrated that some oncogenic lncRNAs could inhibit the apoptosis of cancer cells and promote cell proliferation and migration, as demonstrated in gastric cancer [78]. Furthermore, specific lncRNAs have been implicated in the promotion of chemoresistance and the reprogramming of cellular energy metabolism within malignant cells [79].

### 5.1. lncRNA in RCC Development and Progression

A study investigated LINC01322 RNA expression in RCC patients. Researchers found that LINC01322 expression was significantly elevated in advanced-stage tumors, particularly those located in the left kidney and treated with total nephrectomy. Higher LINC01322 levels also correlated with larger tumor size. Additionally, bioinformatic analysis suggested that LINC01322 may interact with the VHL gene, potentially influencing the tumor microenvironment and contributing to RCC progression. These findings suggest that LINC01322 could serve as a diagnostic marker for high-risk RCC patients, although further studies are needed to confirm its mechanistic link with VHL and to evaluate its potential as a blood-based biomarker [80].

Wilms tumor 1-associated protein (WTAP), a key component of the N6-methyladenosine (m6A) methyltransferase complex, has been found to be significantly upregulated in RCC tissues compared to normal counterparts. WTAP promotes tumor cell proliferation and metastasis both in vitro and in vivo. WTAP facilitates m6A methylation of the long noncoding RNA TEX41, leading to its degradation via YTH N6-methyladenosine RNA binding protein 2 (YTHDF2)-mediated recognition. The loss of TEX41 stabilizes the interaction with SUZ12 polycomb repressive complex 2 subunit (SUZ12), a subunit of the polycomb repressive complex 2 (PRC2), enhancing SUZ12 histone methyltransferase activity and contributing to Histone deacetylase 1 (HDAC1) gene silencing. These findings delineate a novel oncogenic regulatory axis—WTAP/TEX41/SUZ12/HDAC1—in RCC progression, underscoring the critical role of epigenenetic modifications in tumor biology. This axis may offer new insights into RCC pathogenesis and presents potential targets for therapeutic intervention [81].

Marked upregulation of LINC00365 was highlighted in advanced RCC cases and in older patients undergoing total nephrectomy, suggesting its potential as a diagnostic marker for identifying high-risk individuals, particularly among the elderly. Furthermore, higher expression was observed in female patients, revealing its possible use as a gender-specific biomarker. Future studies assessing LINC00365 levels in serum samples are warranted to validate its clinical utility as a non-invasive diagnostic tool [82].

Oxidative stress is caused by an imbalance between the production of reactive oxygen species (ROS) and cells’ antioxidant defenses, and has been associated in the development and progression of several diseases [83,84,85,86,87,88,89]. Although lncRNAs were considered non-translatable, recently it has been discovered that they can encode functional peptides involved in the progression of tumors [90]. In this study, it was discovered that a novel peptide, TCL6-encoded peptide 6148 (TCL6148), was encoded by T-cell leukemia/lymphoma 6 (TCL6). Chen et al. demonstrated that TCL6148 exerts anti-tumor activity by inducing ferroptosis in RCC via the glutamic-oxaloacetic transaminase 1 (GOT1)/GPX4 signaling pathway. TCL6148 promoted the accumulation of Fe^2+^, increased reactive ROS levels, and enhanced lipid peroxidation, thereby increasing RCC cell susceptibility to ferroptosis. Notably, TCL6148 markedly improved the efficacy of sunitinib, suggesting a potential combinational strategy to overcome drug resistance. In vivo studies further confirmed the safety and tumor-suppressive effects of TCL6148. Overall, the results obtained by this research group highlight TCL6148 as a promising therapeutic agent for RCC and offer new perspectives on peptide-based treatments for this malignancy [91].

G protein-coupled receptor class C group 5 member D antisense RNA 1 (GPRC5D-AS1) is a lncRNA that was studied in lung squamous cell carcinoma as biomarker in clinical applications. In RCC, its expression was studied in renal cancer cell line 786-O and in nude mice. Jia et al. showed that after *GPRC5D-AS1* silencing there was an increase in the number of proliferating cells and migrating cells, as well as an important increase in tumor volume in nude mice and higher levels of β-catenin, Ki-67, PCNA and N-cadherin. Results showed an opposite trend for cells that overexpressed *GPRC5D-AS1* [92].

Li et al. revealed that LINC00645 is among the most dysregulated lncRNA between RCC and healthy renal tissue. In this study, they showed that it is involved in RCC development. In particular, it was demonstrated that LINC00645 functions as a tumor suppressor in RCC, inhibiting cell proliferation, migration, and invasion. RNA-binding protein heterogeneous nuclear ribonucleoprotein A2/B1 (HNRNPA2B1) stabilized rho-associated coiled-coil containing protein kinase 1 (ROCK1) mRNA by direct binding. LINC00645 competes for the RRM1 domain of HNRNPA2B1, which is crucial for its interaction with ROCK1 mRNA, thereby promoting ROCK1 mRNA destabilization and reducing its expression. In RCC cells, decreased LINC00645 expression disrupts this interaction, leading to increased ROCK1 levels and enhanced tumor progression. Notably, RCC cells with low LINC00645 expression exhibit heightened sensitivity to the ROCK1 inhibitor Y-27632. These findings suggest that the LINC00645/HNRNPA2B1/ROCK1 axis plays a critical role in RCC progression and presents a potential therapeutic target, especially for patients with reduced LINC00645 expression [93].

The expression of lncRNA HIF1A antisense RNA 2 (HIF1A-AS2), located on chromosome 14, was studied in different tumors, including breast, lung, gastric and bladder cancer. In ccRCC, HIF1A-AS2 is known to promote cell proliferation through the miR-130a-5p/Erb-B2 receptor tyrosine kinase 2 (HER2) (ERBB2) and miR-30a-5p/SRY-box transcription factor 4 (SOX4) pathways. Recent studies have expanded this understanding by demonstrating that HIF1A-AS2 also facilitates ccRCC cell migration via the regulation of hypoxia-inducible factor 1 alpha (HIF1α) mediated by the transcription factor Gli family zinc finger 1 (Gli1). Expression analyses in tumor and normal tissues, supported by public databases and 30 clinical samples, confirmed significant upregulation of HIF1A-AS2 in tumor tissues. Knockdown of HIF1A-AS2 in ccRCC cell lines resulted in marked reductions in cell proliferation and migration. Bioinformatic and experimental approaches revealed a direct interaction between HIF1A-AS2 and Gli1, with HIF1A-AS2 positively regulating Gli1 expression. Gli1, which is overexpressed in ccRCC, in turn modulates HIF1α expression, a well-known oncogene that accumulates due to common VHL mutations in ccRCC. Further experiments demonstrated that Gli1 directly binds the promoter region of HIF1α, enhancing its transcription. Given the central role of HIF1α in lipid metabolism in ccRCC, RNA-seq analyses of HIF1A-AS2 knockdown cells revealed significant alterations in lipid metabolic pathways, with sterol regulatory element binding transcription factor 1 (SREBP1) identified as a potential downstream effector of HIF1A-AS2.

These findings highlight the critical role of HIF1A-AS2 in promoting ccRCC progression through modulation of the Gli1/HIF1α axis and remodeling of lipid metabolism, providing new therapeutic insights [94].

lncRNA osteopetrosis-associated transmembrane protein 1 anti-sense RNA 1 (OSTM1-AS1) was found, in the studies of Chen et al., to be one of the most abundant lncRNAs, and it was highly expressed both in RCC tissue and cell lines. OSTM1-AS1 promoted cell migration and invasion by suppressing miR-491-5p, which normally inhibits matrix metallopeptidase 9 (MMP-9), a key enzyme involved in tumor progression. Silencing OSTM1-AS1 increased miR-491-5p levels, reduced MMP-9 expression, and suppressed RCC cell invasiveness, while reversing these effects by downregulating miR-491-5p or overexpressing MMP-9 restored malignancy. In vivo, OSTM1-AS1 knockdown significantly reduced tumor growth in mice. The OSTM1-AS1/miR-491-5p/MMP-9 axis plays a crucial role in RCC progression and may offer a novel therapeutic target [95].

Vasculogenic mimicry (VM) presents a critical role in the invasion and metastasis of various tumors, including RCC. A recent study identified lncRNA-SERB as significantly overexpressed in RCC tumor cells. Both in vitro and in vivo mouse model analyses demonstrated that lncRNA-SERB enhanced VM formation and consequently promoted tumor cell invasion. LncRNA-SERB acted as an upstream regulator of ERβ by binding to its 3′UTR, thereby increasing ERβ expression. Elevated ERβ, in turn, facilitated VM through the transcriptional activation of zinc finger E-box binding homeobox 1 (ZEB1), a key factor in tumor progression. Therapeutically, the administration of the Food and Drug Administration (FDA)-approved anti-estrogen ICI 182,780 in preclinical models significantly reduced tumor growth and metastasis, underscoring the potential of targeting the lncRNA-SERB/ERβ/ZEB1 axis in RCC. These findings offer novel insights into RCC pathogenesis and present new avenues for therapeutic intervention aimed at disrupting VM-driven tumor progression [96].

Hereditary leiomyomatosis and renal cell carcinoma (HLRCC) is one of the most aggressive RCCs, characterized by germline loss-of-function mutation of fumarate hydratase (FH) [97].

FH-deficient RCC represents a distinct subtype of kidney cancer characterized by disrupted cellular metabolism, although the mechanisms of its metabolic reprogramming remain poorly understood. This study investigated the role of lncRNAs in this context by integrating RNA-sequencing and mass spectrometry on FH-deficient RCC tissues and conducting functional assays using the UOK262 cell line and patient-derived xenograft (PDX) models. The lncRNA MIR4435-2HG was found to be selectively overexpressed in FH-deficient RCC compared to ccRCC. Mechanistically, MIR4435-2HG expression was epigenetically upregulated by fumarate through histone demethylation. Functional studies revealed that MIR4435-2HG enhanced glutamine metabolism by binding to signal transducer and activator of transcription 1 (STAT1), which transcriptionally activated glutaminase 1 (GLS1). Targeting GLS1 with the inhibitor CB-839 significantly reduced tumor growth in vivo. These findings highlight MIR4435-2HG as a key regulator of metabolic remodeling in FH-deficient RCC and propose GLS1 as a promising therapeutic target, with MIR4435-2HG serving as a potential biomarker for treatment response [98].

### 5.2. Interactions and Cross-Talk Between Short and Long Non-Coding RNAs: Mutual Regulation of miRNAs and lncRNAs

Many lncRNAs can act as miRNA sponges, binding mRNA and regulating downstream genes expression. An example is lncRNA DMDRMR, which acts as sponge for miR-378a-5p and upregulates EXH2 and SMAD-specific E3 ubiquitin protein ligase 1 (SMURF1). EZH2 prevents the transcription of DAB2 interacting protein (DAB2IP); meanwhile, SMURF1 regulates DAB2IP degradation. This mechanism activates the vascular endothelial growth factor A (VEGFA)/vascular endothelial growth factor receptor 2 (VEGFR2) pathway, which drives angiogenesis in ccRCC and contributes to sunitinib resistance [99].

lncRNA E2F1 mRNA stabilizing factor (EMS) was found to be overexpressed in sorafenib-resistant RCC tissues and cells. Wang et al. demonstrated that silencing EMS enhanced the effectiveness of sorafenib, reducing cell growth and promoting apoptosis in resistant RCC cells. In animal models, combining EMS knockdown with sorafenib therapy significantly suppressed tumor progression. Mechanistically, EMS acts as a molecular sponge for miR-363-3p, whose levels are reduced in resistant cells, thereby preventing it from downregulating dual-specificity phosphatase 10 (DUSP10), a gene that contributes to drug resistance. Restoring miR-363-3p or suppressing DUSP10 re-sensitized cells to sorafenib. The EMS/miR-363-3p/DUSP10 pathway plays a key role in sorafenib resistance in RCC and may serve as a valuable therapeutic target and biomarker [100].

A recent study by Zhu et al. explored the role of m6A modification in RCC and its interaction with lncRNA LHX1 divergent transcript (LHX1-DT). Low expression of LHX1-DT was found in RCC tissues, and it was related to poor overall survival in RCC patients. Moreover, they demonstrated, using functional assays, that the overexpression of LHX1-DT reduced RCC cell proliferation and invasion. The m6A reader protein insulin-like growth factor 2 mrna binding protein 2 (IGF2BP2), regulated by methyltransferase-like 14 (METTL14), specifically recognized the m6A modification site on LHX1-DT, thereby enhancing its stability. Furthermore, LHX1-DT behaved as a competing endogenous RNA (ceRNA) by sequestering miR-590-5p, leading to the upregulation of programmed cell death 4 (PDCD4), which suppresses RCC cell proliferation and invasion. The authors suggested that LHX1-DT operates as an independent prognostic biomarker for RCC, and the IGF2BP2/LHX1-DT/miR-590-5p/PDCD4 signaling axis constitutes a promising therapeutic target for RCC progression [101].

The role of HOXA transcript at the distal tip (HOTTIP), a lncRNA, was investigated in RCC by Zhang et al. HOTTIP resulted in upregulation in RCC tissues with respect to normal tissues and correlated with a poorer prognosis based on the TCGA database. They demonstrated that HOTTIP functioned as a ceRNA for miR-506. In particular, when downregulating HOTTIP, cell proliferation, migration and invasion were found to reduce [102].

Tian et al. investigated the role of the long non-coding RNA small nucleolar RNA host gene 1 (SNHG1) in regulating autophagy and sunitinib resistance in RCC. Elevated SNHG1 expression was observed in RCC tissues and cell lines and was associated with poor patient prognosis. Functional assays revealed that silencing SNHG1 inhibited RCC cell proliferation, migration, invasion, and autophagy, while also restoring sensitivity to sunitinib treatment. Mechanistically, SNHG1 was shown to bind to the RNA-binding protein polypyrimidine tract binding protein 1 (PTBP1), which in turn upregulated ATG7, a key autophagy-related gene. Through this SNHG1/PTBP1/ATG7 axis, SNHG1 promoted malignant progression and drug resistance in RCC cells [103].

The bond of some lncRNAs with proteins can promote changes in downstream genes. For example, different studies highlighted that the androgen receptor (AR) could be involved in RCC progression and metastasis. Wei et al. discovered that the lncRNA suppressing androgen receptor in renal cell carcinoma (SARCC) binds to and promotes the degradation of the AR protein, resulting in the downregulation of miR-143-3p. This, in turn, suppresses downstream signaling pathways involving Akt, matrix metallopeptidase 13 (MMP-13), K-ras, and phosphorylated extracellular signal-regulated kinase (p-ERK). Moreover, sunitinib has been shown to upregulate lncRNA SARCC expression, thereby enhancing RCC cell sensitivity to the drug and reducing resistance [104].

lncRNA HOTAIR is known to be involved in the proliferation, migration, invasion and inhibition of apoptosis in RCC [105,106]. Furthermore, it was demonstrated that lncRNA HOTAIR competitively binds miR-17-5p and regulates Beclin1 in RCC cells, leading to the activation of cell autophagy and RCC resistance to sunitinib. Indeed, higher levels of autophagy are involved in resistance in cancer cells, and are considered a tolerance mechanism of cells towards tumor therapy [107].

### 5.3. Role of lncRNAs in Resistance to Targeted Therapies

An in vivo study conducted by Pan et al. identified a lncRNA, STX17 divergent transcript (STX17-DT), involved in axitinib resistance in RCC. STX17-DT is overexpressed in axinitib-resistant RCC cells and is related to worse patient outcomes. They demonstrated that STX17-DT enhanced the stability of interferon alpha inducible protein 6 (IFI6) mRNA by interacting with the protein heterogeneous nuclear ribonucleoprotein A1 (hnRNPA1), which reduced mitochondrial ROS levels and inhibited ferroptosis. STX17-DT was also loaded into extracellular vesicles via hnRNPA1, enabling the spread of resistance between cells. Notably, combining axitinib with STX17-DT-targeted siRNA improved treatment effectiveness compared to axitinib alone [108].

LncRNA IGFL2-AS1 is significantly upregulated in sunitinib-resistant RCC and is associated with reduced survival rates in ccRCC patients undergoing sunitinib treatment. IGFL2-AS1 competitively interacts with HNRNPC, a versatile RNA-binding protein, which leads to increased expression of tumor protein p53 inducible nuclear protein 2 (TP53INP2). The elevated levels of TP53INP2 stimulate autophagy, contributing to the development of sunitinib resistance in RCC cells. Additionally, IGFL2-AS1 can be encapsulated within exosomes, facilitating the transfer of sunitinib resistance to neighboring RCC cells [109].

It is known that lncRNA SNHG12 has a role in tumor development. Liu et al. investigated the role of SNHG12 in RCC tissue sample and sunitinib-resistant cells, where it resulted in upregulation and was related to a poor clinical prognosis. lncRNA SNHG12 interacts with the transcription factor specificity protein 1 (Sp1), inhibiting its deubiquitination and thereby increasing Sp1 protein stability. Moreover, SNHG12 promotes the binding of Sp1 to cell division cycle-associated protein-3 (CDCA3) promoter, upregulating it [110].

The release of exosomes with lncRNAs can be considered a mechanism to induce drug resistance in RCC cells. LncRNA activated in RCC with sunitinib resistance (ARSR) promoted resistance to sunitinib in RCC cells binding miR-34/miR-449 and then upregulating AXL receptor tyrosine kinase (AXL) and c-Met [111]. Meanwhile, LncARSR can be encapsulated within exosomes, facilitating the transfer of drug resistance from sunitinib-resistant cells to sensitive ones [111].

In another study, exosomes isolated from sunitinib-sensitive and -resistant RCC cells were characterized by transmission electron microscopy and Western blot analysis. Co-culture experiments revealed that exosomes from sunitinib-resistant RCC cells (R-exos) enhanced proliferation and upregulated key proliferative markers, cyclin D1 (CCND1) and proliferating cell nuclear antigen (PCNA), while suppressing apoptosis and reducing Bcl-2-associated X protein (Bax) and Caspase-3 expression by delivering the long non-coding RNA SNHG16. In resistant cell-derived xenograft models, R-exos promoted tumor growth, whereas SNHG16 knockdown significantly inhibited tumor progression. Mechanistically, SNHG16 modulated trophinin-associated protein (TROAP) expression by sponging miR-106a-5p, with miR-106a-5p inhibition or TROAP overexpression reversing the anti-tumor effects of SNHG16 silencing. These findings indicate that the SNHG16/miR-106a-5p/TROAP axis plays a pivotal role in mediating sunitinib resistance and tumor aggressiveness in RCC, highlighting this pathway as a potential therapeutic target for patients undergoing sunitinib treatment [112].

lncRNA leucine-rich repeat containing 75 A-antisense RNA1 (LRRC75A-AS1) was identified as a potential lncRNA that could predict the efficacy of immune checkpoint inhibitor therapy and cancer progression in RCC. Tokunaga et al. showed that LRRC75A-AS1 was upregulated in ccRCC tissues compared with in adjacent normal tissues, and it was related to a poor outcome in patients with RCC. LRRC75A-AS1 regulated cell proliferation and invasion; indeed, its knockdown reduced tumorigenesis [113].

lncRNA Polo-like kinase 1 substrate 1 (PLK1S1) was found to be upregulated in RCC and was related to a poor prognosis. lncRNA PLK1S1 promoted sorafenib resistance in RCC cells through the regulation of C-X-C motif chemokine receptors 5 (CXCR5) sponging miR-653 [114].

Xu et al. discovered a novel lncRNA, termed sorafenib resistance-associated lncRNA in RCC (SRLR), which is overexpressed in sorafenib-resistant RCC cells. SRLR directly interacted with the nuclear transcription factor nuclear factor kappa B (NF-κB), promoting interleukin 6 (IL-6) transcription and activating the signal transducer and activator of transcription (STAT3) signaling pathway, ultimately contributing to sorafenib resistance [115]. Another lncRNA, KIF9-AS1, functions by sponging miR-497-5p, thereby activating the TGF-β and autophagy signaling pathways, and also enhancing RCC cell resistance to sorafenib [116].

lncRNA nuclear-enriched abundant transcript 1 (NEAT1) is upregulated in various human cancers, among which are RCC. In malignant cells, NEAT1 modulated target gene expression, promoting cell proliferation, migration, and invasion while inhibiting apoptosis, primarily through its interaction with miRNAs. In RCC, elevated NEAT1 levels correlated with tumor progression and poor patient survival. Mechanistically, NEAT1 enhanced epithelial–mesenchymal transition and contributed to sorafenib resistance by regulating c-Met expression through competitive binding with miR-34a [117].

**Table 3:** Biological and clinical relevance of lncRNAs in RCC.

lncRNA	Biological and Clinical Relevance	Expression Levels in RCC	Ref.
LINC01322	Interacts with VHL gene and contributes to RCC progression	High expression in advanced stages	[80]
TEX41	Facilitates m6A methylation and degradation; loss stabilizes interaction with SUZ12 enhancing HDAC1 gene silencing, promoting RCC progression.	Downregulated (due to degradation)	[81]
LINC00365	Diagnostic marker; higher expression in advanced RCC, elderly, and female patients; potential gender-specific biomarker	Upregulated	[82]
TCL6	Peptide TCL6148 induces ferroptosis via GOT1/GPX4 pathway, promoting Fe^2+^ accumulation and enhancing sensitivity to sunitinib	Downregulated in RCC and correlated with poor prognosis	[91]
GPRC5D-AS1	Suppresses proliferation and migration when overexpressed; silencing increases tumor growth and proliferation markers	Downregulated in RCC cell lines	[92]
LINC00645	Tumor suppressor; inhibits proliferation, migration, invasion; destabilizes ROCK1 mRNA via HNRNPA2B1 interaction; low levels promote tumor progression	Downregulated	[93]
HIF1A-AS2	Promotes proliferation and migration via Gli1/HIF1α axis; regulates lipid metabolism; knockdown reduces tumor growth	Upregulated	[94]
OSTM1-AS1	Promotes migration and invasion by suppressing miR-491-5p, leading to increased MMP-9; knockdown reduces tumor growth	Upregulated	[95]
lncRNA-SERB	Promotes tumor cell invasion enhancing VM formation; increases Erβ expression	Upregulated	[96]
MIR4435-2HG	Upregulated by fumarate through histone demethylation; enhances glutamine metabolism binding STAT1 that activates GLS1 → inhibiting GLS1 with CB-839 reduces tumor growth in vivo	Upregulated in FH-deficient RCC compared to ccRCC	[98]
DMDRMR	Triggers VEGFA/VEGFR2 signaling by sponging miR-378a-5p, boosting EZH2 and SMURF1 to suppress DAB2IP, promoting angiogenesis and sunitinib resistance	Upregulated in ccRCC	[99]
EMS	Sponges miR-363-3p, upregulates DUSP10, promotes sorafenib resistance and tumor growth	Upregulated in sorafenib-resistant RCC tissues and cells	[100]
LHX1-DT	Stabilized by m6A reader IGF2BP2; sponges miR-590-5p to upregulate PDCD4, suppressing proliferation and invasion	Low expression; associated with poor survival	[101]
HOTTIP	Acts as ceRNA for miR-506; promotes proliferation, migration and invasion	Upregulated; correlates with poor prognosis	[102]
SNHG1	Binds PTBP1 to upregulate ATG7, enhancing autophagy and sunitinib resistance	Upregulated in RCC and sunitinib-resistant cells	[103]
SARCC	Binds and degrades androgen receptor (AR), downregulates miR-143-3p, suppresses Akt/MMP-13/K-Ras/p-ERK pathways; increases sunitinib sensitivity	Upregulated by sunitinib	[104]
HOTAIR	Sponges miR-17-5p to regulate Beclin1, activates autophagy, contributes to proliferation and sunitinib resistance	Upregulated in RCC	[105,106,107]
STX17-DT	Enhances IFI6 mRNA stability via hnRNPA1, reduces ROS and ferroptosis, promotes axitinib resistance	Upregulated in axitinib-resistant RCC	[108]
IGFL2-AS1	Interacts with HNRNPC to upregulate TP53INP2, promotes autophagy and sunitinib resistance; transferred via exosomes	Upregulated in sunitinib-resistant RCC	[109]
SNHG12	Binds Sp1 to prevent deubiquitination, promotes CDCA3 expression, linked to poor prognosis	Upregulated in RCC and sunitinib-resistant cells	[110]
ARSR	Sponges miR-34/miR-449, upregulates AXL and c-Met, transfers resistance via exosomes	Upregulated in sunitinib-resistant RCC	[111]
SNHG16	Delivered by exosomes; sponges miR-106a-5p, upregulates TROAP, promotes tumor growth and sunitinib resistance	Upregulated in sunitinib-resistant RCC	[112]
LRRC75A-AS1	Regulates proliferation and invasion, predicts poor outcomes and immune checkpoint inhibitor efficacy	Upregulated in ccRCC tissues	[113]
PLK1S1	Promotes sorafenib resistance via CXCR5 by sponging miR-653	Upregulated in RCC	[114]
SRLR	Binds NF-κB, promotes IL-6 transcription and STAT3 signaling, inducing sorafenib resistance	Upregulated in sorafenib-resistant RCC	[115]
KIF9-AS1	Sponges miR-497-5p, activates TGF-β and autophagy pathways, enhances sorafenib resistance	Upregulated in sorafenib-resistant RCC	[116]
NEAT1	Sponges miR-34a, upregulates c-Met, promotes epithelial–mesenchymal transition (EMT), proliferation, invasion, sorafenib resistance	Upregulated in RCC	[117]

### 5.4. Targeting of Non-Coding RNAs

The recognition of ncRNAs as key regulators of gene expression has prompted the development of therapeutic strategies aimed at modulating their activity. For miRNAs, two main approaches have been established: inhibition of oncogenic miRNAs using antisense oligonucleotides, including antagomiRs and locked nucleic acid (LNA)-modified inhibitors, and restoration of tumor-suppressive miRNAs through the delivery of synthetic miRNA mimics [118]. These strategies enable sequence-specific modulation of miRNA function and have demonstrated biological activity in multiple preclinical models.

LncRNAs and circRNAs, which often exert their functions through interactions with DNA, RNA, or proteins, can be targeted using antisense oligonucleotides or small interfering RNAs (siRNAs) designed to promote transcript degradation or disrupt functional domains [119,120]. In addition, CRISPR/Cas-based approaches have been explored to selectively interfere with ncRNA genomic loci or regulatory regions, although their therapeutic application remains largely experimental. CircRNAs, due to their covalently closed structure and high stability, represent particularly attractive targets but also pose technical challenges for efficient and specific silencing.

One of the major limitations of ncRNA-based therapies is effective and selective delivery. To overcome this barrier, several delivery platforms are under investigation, including lipid- and polymer-based nanoparticles, chemically conjugated oligonucleotides, and extracellular vesicle-based systems, which aim to improve stability, biodistribution, and cellular uptake [121]. Although most ncRNA-targeting strategies are still in preclinical development, accumulating evidence suggests that modulation of miRNA, lncRNA, and circRNA activity may represent a promising therapeutic avenue. Further advances in delivery technologies and a deeper understanding of ncRNA biology will be essential for the successful clinical translation of ncRNA-based therapeutic strategies.

## 6. Conclusions

RCC continues to represent a major clinical challenge because it often develops without symptoms, frequently presents with metastatic spread at diagnosis, and shows limited responsiveness to conventional treatments. Within this context, ncRNAs, especially miRNAs, lncRNAs, and circRNAs, have gained attention as crucial regulatory molecules involved in tumor initiation, disease progression, and resistance to therapy.

An increasing body of evidence indicates that ncRNAs not only act as oncogenic drivers or tumor suppressors, but also hold substantial value as diagnostic, prognostic, and predictive biomarkers. Some ncRNAs are able to distinguish among RCC histological subtypes, whereas others correlate with responsiveness or resistance to targeted agents such as sunitinib, sorafenib, and axitinib. In addition, the complex interplay between lncRNAs and miRNAs, together with the expanding functional relevance of circRNAs, underscores the multilayered nature of gene regulation in RCC. These findings also point to new therapeutic opportunities, including strategies aimed at inhibiting or modulating ncRNA-dependent signaling networks. Overall, improving our understanding of ncRNA-mediated mechanisms in RCC may facilitate earlier detection, refine patient stratification, and promote the development of more personalized treatment strategies.

From a clinical and translational perspective, several ncRNAs discussed in this review emerge as particularly relevant biomarker candidates and therapeutic targets in RCC. Circulating and tissue-derived miRNAs such as miR-21, miR-210, miR-155, and miR-301a-3p show consistent associations with RCC subtype discrimination, disease progression, and patient outcome, supporting their potential use as diagnostic or prognostic biomarkers. Among lncRNAs, molecules such as ARSR, SNHG1, HOTAIR, and STX17-DT actively drive resistance to targeted therapies, while others, including LINC00645 and GAS5, exhibit tumor-suppressive functions, highlighting their therapeutic relevance. In addition, emerging circRNAs such as circASAP1, circGRAMD4, circPDHK1, and circAGAP1 regulate key processes including ferroptosis, immune evasion, and drug sensitivity, positioning them as novel targets for therapeutic intervention. Together, these ncRNAs represent a restricted set of high-priority candidates for future validation studies aimed at improving biomarker development and ncRNA-based therapeutic strategies in renal cell carcinoma. Further translational and clinical investigations will be required to fully exploit ncRNAs as actionable targets in renal cancer management.

## Figures and Tables

**Table 1:** Biological and clinical relevance of miRNAs in RCC.

miRNA	Biological and Clinical Relevance	Expression Levels in RCC	Ref.
miR-210/miR-221	Hypoxia-induced; used in subtype classification (ccRCC vs. papillary); linked to progression	Upregulated in ccRCC	[48]
miR-200c/miR-139-5p	Distinguishes oncocytoma from chromophobe RCC; downregulated after surgery	Downregulated post-surgery	[48]
miR-31/miR-126	Differentiate ccRCC from papillary tumors	Not specified	[48]
miR-424 and miR-203	Discriminates ccRCC from papillary	Upregulated in ccRCC	[49]
miR155, miR-21 and miR210	Induced by hypoxia	Upregulated in ccRCC	[50]
miR-25-3p, miR-126-5p, miR-200c-3p, and miR-301a-3p	Not specified in function	downregulated in ccRCC after surgery	[51]
miR-1293	Potential biomarker for disease progression	Upregulated in ccRCC after surgery	[51]
miR-301a-3p	Potential role in metastasis	Upregulated in patients with metastatic disease	[51]
miR-223, miR-365–2, miR-21, miR-18a, miR-183, miR-335, miR-149, miR-9–2, miR-365–1, miR-130b, miR-9–1, miR-625, and miR-146b	Not specified	Negative association with survival	[52]
miR-584, miR-10b, miR-27b, miR-769, miR-181a-2, miR-23b, miR-204, miR-24–1, and miR-139	Not specified	Positively associated with better survival outcomes	[52]
miR-497		Low expression is associated with more aggressive stages	[53]
miR-21-5p, miR-223-3p, and miR-365a-3p		Overexpression is associated with worse survival outcomes in ccRCC	[54]
miR-99a-3p	Targets PTEN	Upregulated in sunitinib-resistant ccRCC	[61]
miR-96-5p and miR-29b-3p	Targets THBS1	Upregulated in ccRCC, downregulates THBS1, contributing to sunitinib resistance in ccRCC	[62]
miR-130b	Inhibits PTEN	Upregulated in RCC cell lines	[63]
miR-885-5p	- Regulates PLIN3, which is involved in lipid droplet formation to protect against oxidative stress and lipotoxic damage - Suppressed by GATA1 downregulation - Its suppression contributes to sunitinib resistance in ccRCC	Significantly reduced after sunitinib treatment; associated with poor disease progression and drug resistance	[64]
miR-31-5p	Promotes sorafenib resistance by being transferred via EVs from resistant to sensitive cells; suppresses MLH1, a drug sensitivity gene	Upregulated in EVs from sorafenib-resistant RCC cells and in patient plasma during disease progression	[65]
miR-2355-5p	Oncogenic miRNA regulated by HIF-2α in VHL-deficient ccRCC; targets tumor suppressors (ACO1, BTG2, CMTM4, SLIT2, WDFY2); promotes angiogenesis and growth	Significantly upregulated in ccRCC tissues, cell lines, and plasma of patients	[66]
miR-210-3p	Enhances doxorubicin sensitivity by targeting ABCC1/3′UTR, reducing expression of MRP1, an efflux transporter involved in chemoresistance	Downregulated in doxorubicin-resistant RCC cells (Caki-2/DOX)	[67]
miR-124	Induces cisplatin resistance by targeting CAPN4, protecting CNOT3 from degradation, thereby suppressing necroptosis, a form of programmed cell death	Upregulated in RCC cells following cisplatin treatment	[68]

**Table 2:** Biological and clinical relevance of circRNAs in RCC.

circRNA	Biological and Clinical Relevance	Expression Levels in RCC	Ref.
circASAP1	Correlated with ccRCC cell viability, invasion and metastasis. Knockdown suppresses RCC progression and metastasis by promoting ferroptosis.	Upregulated in ccRCC	[76]
circGRAMD4	Its loss correlated with a marked suppression of RCC proliferation. It binds RBM4 protein to stabilize NBR1 mRNA, enhancing MHC-I degradation via autophagy and impairing CD8+ T cell function → immune evasion.	Upregulated in RCC: high levels correlated with a poor patient prognosis	[74]
Circ_0003520	Oncogene: promotes proliferation, migration, invasion, and angiogenesis by sponging miR-205-5p → upregulates CUL4B.	Upregulated in ccRCC	[73]
circPDHK1	Drives proliferation and migration; therapeutic inhibition via AS1411/LNP-siRNA blocks mTOR-AKT signaling.	Upregulated in ccRCC; targeted by siRNA delivery system for therapy	[77]
circAGAP1	Enhances sunitinib sensitivity by sponging miR-149-5p, miR-455-5p, and miR-15a-5p, modulating PDGFR expression.	Upregulated in sunitinib-sensitive ccRCC cells; correlates with better drug response	[75]

## Data Availability

No new data were created or analyzed in this study.

## Abbreviations

The following abbreviations are used in this manuscript:

3′UTR	3′ untranslated region
ABCC1	ATP-binding cassette subfamily C member 1
ACO1	Aconitase 1
Akt	Protein kinase B
AR	Androgen receptor
ASAP1	ArfGAP with SH3 domain, ankyrin repeat and PH domain 1
ATG7	Autophagy-related 7
ATG14	Autophagy-related 14
AXL	AXL receptor tyrosine kinase
BAP1	BRCA1-associated protein 1
Bak1	BCL2 antagonist/killer 1
Bax	Bcl-2-associated X protein
Bcl-2	B-cell lymphoma 2
BTG2	BTG anti-proliferation factor 2
CAPN4	Calpain 4
ccRCC	Clear cell renal cell carcinoma
CD8+	Cluster of differentiation 8 positive (T lymphocytes)
CDCA3	Cell division cycle-associated 3
ceRNA	Competing endogenous RNA
chRCC	Chromophobe renal cell carcinoma
circRNA(s)	Circular RNA(s)
CMTM4	CKLF-like MARVEL transmembrane domain-containing 4
CNOT3	CCR4-NOT transcription complex subunit 3
CRISPR/Cas9	Clustered regularly interspaced short palindromic repeats/CRISPR-associated protein 9
CUL4B	Cullin 4B
CXCR5	C-X-C motif chemokine receptor 5
DAB2IP	DAB2-interacting protein
DHFR	Dihydrofolate reductase
DMDRMR	(lncRNA) DMDRMR
DNA	Deoxyribonucleic acid
DOX	Doxorubicin
DUSP10	Dual-specificity phosphatase 10
EGFR	Epidermal growth factor receptor
EIF3J-DT	EIF3J divergent transcript
EMS	E2F1 mRNA stabilizing factor
EMT	Epithelial–mesenchymal transition
ERBB2	Erb-B2 receptor tyrosine kinase 2 (HER2)
ERK	Extracellular signal-regulated kinase
ERβ	Estrogen receptor beta
EV(s)	Extracellular vesicle(s)
EZH2	Enhancer of zeste homolog 2
FDA	Food and Drug Administration
Fe^2+^	Ferrous ion
FH	Fumarate hydratase
GOT1	Glutamic-oxaloacetic transaminase 1
GPX4	Glutathione peroxidase 4
GLS1	Glutaminase 1
Gli1	GLI family zinc finger 1
GPRC5D-AS1	G protein-coupled receptor class C group 5 member D antisense RNA 1
GATA1	GATA-binding protein 1
GSTM2	Glutathione S-transferase mu 2
HDAC1	Histone deacetylase 1
HEY1	Hes-related family bHLH transcription factor with YRPW motif 1
HIF	Hypoxia-inducible factor
HIF-2α	Hypoxia-inducible factor 2 alpha
HIF1α	Hypoxia-inducible factor 1 alpha
HIF1A-AS2	HIF1A antisense RNA 2
HLRCC	Hereditary leiomyomatosis and renal cell carcinoma
HNRNPC	Heterogeneous nuclear ribonucleoprotein C
HNRNPA1	Heterogeneous nuclear ribonucleoprotein A1
HNRNPA2B1	Heterogeneous nuclear ribonucleoprotein A2/B1
HOTTIP	HOXA transcript at the distal tip
HOTAIR	HOX transcript antisense RNA
ICB	Immune checkpoint blockade
IFI6	Interferon alpha inducible protein 6
IGF2BP2	Insulin-like growth factor 2 mRNA binding protein 2
IGFL2-AS1	IGF-like family member 2 antisense RNA 1
IL-6	Interleukin 6
lncRNA(s)	Long non-coding RNA(s)
LHX1-DT	LHX1 divergent transcript
LINC	Long intergenic non-protein coding RNA
LRRC75A-AS1	Leucine-rich repeat containing 75A antisense RNA 1
MALAT1	Metastasis-associated lung adenocarcinoma transcript 1
m6A	N6-methyladenosine
MEG3	Maternally expressed gene 3
METTL14	Methyltransferase like 14
MHC-I	Major histocompatibility complex class I
miRNA(s)	MicroRNA(s)
MLH1	MutL homolog 1
MMP-9	Matrix metallopeptidase 9
MMP-13	Matrix metallopeptidase 13
MRP1	Multidrug resistance-associated protein 1
mRNA	Messenger RNA
mTOR	Mechanistic target of rapamycin
MYC	MYC proto-oncogene
ncRNA(s)	Non-coding RNA(s)
NEAT1	Nuclear paraspeckle assembly transcript 1
NF-κB	Nuclear factor kappa B
NRF2	Nuclear factor erythroid 2-related factor 2
OSTM1-AS1	Osteopetrosis-associated transmembrane protein 1 antisense RNA 1
p-ERK	Phosphorylated ERK
PBRM1	Polybromo 1
PCNA	Proliferating cell nuclear antigen
PDCD4	Programmed cell death 4
PDGFR	Platelet-derived growth factor receptor
PDPK1	3-phosphoinositide dependent protein kinase 1
PDX	Patient-derived xenograft
PLIN3	Perilipin 3
PLK1S1	Polo-like kinase 1 substrate 1
pRCC	Papillary renal cell carcinoma
PRC2	Polycomb repressive complex 2
PTBP1	Polypyrimidine tract binding protein 1
PTEN	Phosphatase and tensin homolog
PVT1	Plasmacytoma variant translocation 1
qRT-PCR	Quantitative reverse transcription–polymerase chain reaction
RAS	Rat sarcoma proto-oncogene
RBM4	RNA binding motif protein 4
RCC	Renal cell carcinoma
RRM2	Ribonucleotide reductase regulatory subunit M2
RBP(s)	RNA-binding protein(s)
ROCK1	Rho-associated coiled-coil containing protein kinase 1
ROS	Reactive oxygen species
RRMs	RNA recognition motifs
SARCC	Suppressing androgen receptor in renal cell carcinoma
SETD2	SET domain containing 2, histone lysine methyltransferase
shRNA	Short hairpin RNA
siRNA	Small interfering RNA
SLIT2	Slit guidance ligand 2
SMURF1	SMAD specific E3 ubiquitin protein ligase 1
SNHG1	Small nucleolar RNA host gene 1
SNHG12	Small nucleolar RNA host gene 12
SNHG16	Small nucleolar RNA host gene 16
SOX4	SRY-box transcription factor 4
Sp1	Specificity protein 1
SREBP1	Sterol regulatory element binding transcription factor 1
SRLR	Sorafenib resistance-associated lncRNA in RCC
STAT1	Signal transducer and activator of transcription 1
STAT3	Signal transducer and activator of transcription 3
STX17-DT	STX17 divergent transcript
SUZ12	SUZ12 polycomb repressive complex 2 subunit
TCGA	The Cancer Genome Atlas
TCL6	T-cell leukemia/lymphoma 6
TCL6148	TCL6-encoded peptide 6148
TGFβ	Transforming growth factor beta
THBS1	Thrombospondin 1
TKI(s)	Tyrosine kinase inhibitor(s)
TP53	Tumor protein p53
TP53INP2	Tumor protein p53 inducible nuclear protein 2
TROAP	Trophinin-associated protein
TS	Thymidylate synthase
ubiquitin–proteasome	Ubiquitin–proteasome degradation pathway
VEGF	Vascular endothelial growth factor
VEGFA	Vascular endothelial growth factor A
VEGFR2	Vascular endothelial growth factor receptor 2
VHL	Von Hippel–Lindau
VM	Vasculogenic mimicry
WDFY2	WD repeat and FYVE domain containing 2
Wnt	Wingless-related integration site
WTAP	Wilms tumor 1-associated protein
YTHDF2	YTH N6-methyladenosine RNA binding protein 2
ZEB1	Zinc finger E-box binding homeobox 1
